# Impact of Mating Methods and Semen Preservation on Reproductive and Growth Performances in Palestinian Assaf Sheep

**DOI:** 10.3390/biology14010080

**Published:** 2025-01-16

**Authors:** Wael Halaweh, Samia Khnissi, Ikram Ben Souf, Muayad Salman, Naceur M’Hamdi

**Affiliations:** 1 Research Laboratory of Ecosystems and Aquatic Resources, UR03AGRO, National Agronomic Institute of Tunisia, University of Carthage, Carthage 1054, Tunisia; wael_halawa73@yahoo.com (W.H.); bensoufikram2@gmail.com (I.B.S.); moayednas@yahoo.com (M.S.); 2 Laboratory of Animal and Forage Production, National Institute of Agronomic Research of Tunisia (INRAT), University of Carthage, Tunis 1054, Tunisia; mleilsamia@live.fr

**Keywords:** artificial insemination, natural mating, fertility, lambs’ performances, reproductive biotechnologies

## Abstract

As global demand for sheep products increases, improving reproductive efficiency and lamb growth performance has become a priority for sheep farmers. Artificial insemination (AI) offers several advantages over natural mating, including improved genetic selection and disease control. This study contributes to understanding the impact of different mating methods and semen preservation techniques on reproductive performance and growth traits in Palestinian Assaf sheep under local breeding conditions. The research included 123 adult Assaf ewes from two farms, which were hormonally synchronized and divided into three groups for different reproductive biotechnologies. Group 1 (G1) comprised 50 ewes inseminated with frozen semen straws, while Group 2 (G2) had 37 ewes inseminated with fresh semen from genetically superior rams. Both G1 and G2 used 0.5 mL straws containing 250 million sperm, with insemination occurring 48 h post sponge removal and eCG hormone injection. Group 3 (G3) consisted of 36 ewes that underwent natural mating at a ratio of 1 ram to 6 ewes. The study revealed that insemination methods significantly affected fertility rates (45.4, 61.1, and 71.9% for G1, G2, and G3, respectively; *p* < 0.05). Artificial insemination notably enhanced lamb performance indicators (*p* < 0.05), although it did not significantly influence prolific lambing (*p* > 0.05). Prolific lambing significantly impacted birth weight, weaning weight, and average daily gain at 60 days across all groups (*p* < 0.05), but not the average daily gain or final weight at 180 days (*p* > 0.05). The management system significantly affected birth and weaning weights at 60 days (*p* < 0.05), but not the final weight at 180 days or prolific lambing (*p* > 0.05). Lamb sex significantly influenced average daily gains at 60 and 180 days, as well as final weight at 180 days (*p* < 0.05). The findings suggest that, while artificial insemination with semen from elite rams may reduce fertility rates, it significantly improves lamb performance rates. In conclusion, our study demonstrates that, while natural mating offers higher fertility rates, AI with fresh semen can be a valuable tool for improving lamb growth performance in Palestinian Assaf sheep. These findings provide valuable insights for sheep farmers in the region to optimize reproductive strategies and enhance lamb production

## Simple Summary

As global demand for sheep products such as meat, wool, and milk grows, maximizing reproductive function becomes increasingly important. Traditional, natural mating has drawbacks, such as variable conception rates, disease transmission, and reduced genetic variety. Artificial insemination (AI), when paired with efficient semen storage techniques, provides a solution by improving breeding efficiency, permitting genetic improvement, and ensuring year-round reproduction. In this study, natural mating was compared with artificial insemination (AI), especially under different semen preservation conditions. It was concluded that natural mating provided the highest fertility rates (71.9%), while artificial insemination (AI) with fresh semen had higher fertility rates (61.1%) than frozen semen (45.4%). Despite lower fertility, AI considerably enhanced lamb growth performance, with birth and weaning weights showing the greatest effect. Prolific lambing, management strategies, and lamb sex all had an impact on early growth and final weight results. Overall, this study demonstrates that, while natural mating offers higher fertility rates, AI, especially with fresh semen, can be a valuable tool for improving reproductive efficiency and lamb growth performance in Palestinian Assaf sheep. These findings have important implications for sheep farmers in the region, as they provide valuable insights into optimizing reproductive strategies and improving lamb production

## 1. Introduction

Livestock plays a vital role in Palestine’s agricultural sector, making significant contributions to the rural economy. This sector is essential for agricultural income, with small ruminants like sheep and goats serving as its backbone. In the West Bank and Gaza Strip, there are around 972,000 sheep and goats, along with 39,000 cattle, highlighting the importance of livestock as a primary source of livelihood for many rural households. Additionally, this sector not only provides crucial income but also enhances food security and economic stability in economically disadvantaged areas [1].

According to the Food and Agriculture Organization (FAO), small ruminant production contributes significantly to the overall agricultural income in Palestine, accounting for up to 46% of the total revenue [2]. However, the high cost of imported commercial fodder as a replacement, recurrent drought, and the outbreak of animal diseases have created obstacles to developing the livestock sector in Palestine [3]. Notably, the absence of a robust artificial insemination (AI) management system exacerbates these issues, resulting in inconsistent services for farmers and ultimately leading to a decline in livestock numbers within the region.

Breeding biotechnologies are essential for genetic improvement and biodiversity promotion, as shown by the Assaf breed’s ability to enhance livestock productivity. Successful breeding of Assaf through Booroola gene introgression increased prolificacy [4]. By utilizing artificial insemination (AI) and advanced semen preservation techniques, farmers can access superior genetic material, facilitating the propagation of desirable traits such as increased fertility, enhanced growth rates, and improved disease resistance in livestock. These technologies enable the strategic selection of breeding stock, introducing high-quality genetics into herds, ultimately leading to more productive and resilient animal populations [5]. Proper application of reproductive technologies enables faster advances in genetic gains, producing more resource-use-efficient livestock and better adapted to their environments [6].

Various studies have reported fecundity rates associated with different artificial insemination (AI) techniques in sheep. Vaginal AI can achieve pregnancy rates of approximately 55%, comparable to natural mating [4,7]. Cervical AI has been shown to yield fertility rates ranging from 40% to 70%, particularly with the use of chilled semen [8,9,10]. Laparoscopic AI often results in higher pregnancy rates than other methods, with studies indicating successful outcomes [4,11,12]. Specifically, for Assaf sheep, research highlights that AI techniques can lead to an average litter size of about 1.57 lambs per ewe, demonstrating the effectiveness of these reproductive technologies in enhancing fertility [13,14].

The Palestinian government recognizes the importance of enhancing livestock production, particularly small ruminants, and has prioritized the development of its livestock sector within its agricultural strategic policies [14]. As part of this endeavor, the implementation of reproductive technologies, including artificial insemination (AI), has proven to be a highly efficient approach to augmenting livestock productivity.

AI has played a significant role in the genetic improvement of sheep, serving as a crucial tool in this process. Notably, countries such as Australia, France, Spain, and Canada have widely adopted AI techniques, with annual insemination rates reaching hundreds of thousands of sheep [15]. The utilization of AI has the potential to enhance the genetic improvement of livestock by promoting the widespread use of highly productive male animals [16,17]. By applying AI technologies, advanced breeding techniques, and genetic selection algorithms, the agricultural industry can optimize the breeding process and ensure the dissemination of desirable traits in livestock populations [18]. However, AI is recognized as a first-generation biotechnology that yields slow genetic progress due to its reliance on the best-performing males. In contrast, second-generation biotechnologies, such as Multiple Ovulation Embryo Transfer (MOET) and In Vitro Fertilization (IVF), focus on female genetics, facilitating rapid advancements in genetic improvement. The transformative potential of these advanced techniques, including embryo sexing and cloning, can significantly enhance breeding outcomes [18]. This approach enables the efficient propagation of superior genetic characteristics, ultimately leading to improved livestock productivity and overall quality [19,20].

The Assaf breed, a cross between Improved Awassi and East Friesian sheep, showcases significant growth and productive traits vital for sheep farming in Palestine. Assaf lambs excel in growth with a high weaning weight of 18.5 kg and a pre-weaning average daily gain of 0.238 kg/day. Ewes average 1.60 lambs per lambing, indicating moderate prolificacy, while the favorable Kleiber ratio of 0.026 reflects efficient feed conversion. In the West Bank, Palestine, Assaf ewes exhibited an average total milk yield of 159.4 kg over a lactation period of approximately 115.4 days, with specific production reaching 167.9 kg by 120 days and 197.0 kg by 150 days, while also having an average litter size of 1.35 lambs per ewe at lambing and a lambing interval of 294.4 days [21,22].

The uniqueness of our study lies in its focus on the Assaf breed in Palestine, which is subject to specific environmental, genetic, and management factors that may influence reproductive outcomes differently compared to other reported studies. We acknowledge that reproductive results from natural mating and artificial insemination (AI) using fresh and cryopreserved semen have been reported in the literature. Natural mating in German Holstein cows resulted in the highest increases in vaginal and uterine blood flow, while AI and intravaginal deposition of raw semen or seminal plasma also enhanced blood flow but to a lesser extent. In dromedary camels, natural mating yielded the highest pregnancy rates, with AI using whole undiluted ejaculates performing well when timed with ovulation. Alpine goats showed higher pregnancy and lower mortality rates with natural mating compared to AI with frozen buck semen, although both methods produced similar birth weights. Additionally, laparoscopic artificial insemination with frozen-thawed semen in Zandi ewes resulted in higher pregnancy rates compared to trans-cervical and vaginal methods, with both soybean lecithin and egg yolk extenders being effective for sperm preservation [23,24].

However, our study aimed to provide region-specific insights into the reproductive performance and lamb growth of Palestinian Assaf sheep, which have not been extensively studied under the specific breeding conditions prevalent in Palestine. The primary objective of our study was to investigate the distinctive reproductive performance and growth outcomes of Palestinian Assaf sheep within local breeding environments, utilizing alternatives to natural mating insemination techniques and ram semen storage strategies.

## 2. Materials and Methods

### 2.1. Experiment Area

The research was conducted on two farms situated in Nablus, West Bank, Palestine: 32°13′16.0″ N 35°15′15.8″ E, and Tubas, West Bank, Palestine: 32°19′12.0″ N 35°22′10.9″ E, during the spring of 2022 (20 March to 21 June 2022). Characterized by a moderate climate and an annual rainfall of approximately 640 mm, this region is predominantly agricultural land. Notably, Nablus is nestled within a mountainous terrain, whereas Tubas lies in a valley, both offering unique perspectives of the Jordan Valley.

In Palestine, the breeding season for sheep generally occurs “in season” from late July to early December. During this time, the ewes naturally come into estrus, making it the optimal period for breeding. The “out of season” period typically spans from January to June, when the ewes are less likely to naturally enter estrus. During this time, farmers may use hormonal treatments, such as eCG (Folligon, Intervet, The Netherlands), to induce estrus and facilitate breeding outside the natural breeding season.

### 2.2. Animal Management

A total of 123 adult lactating Assaf ewes aged between 2 and 6 years were randomly selected and divided into three distinct groups for the study. Group 1 (G1) included 50 ewes that underwent artificial insemination with frozen semen. Group 2 (G2) consisted of 37 ewes inseminated with fresh semen. Meanwhile, Group 3 (G3) comprised 36 ewes that engaged in natural mating with farm rams at a ratio of 1 ram to 6 ewes. The semen, both frozen and fresh, was obtained from elite Assaf rams at the AI center of Beit Qad Station in Jenin, Palestine (coordinates: 32.4590° N, 35.3000° E). The Assaf ram, a crossbreed of the Awassi and German East Friesian breeds, is known for its genetic superiority. At 2 years of age, these rams weigh between 110 and 120 kg and are fed a daily ration consisting of 2 kg concentrated mix with 18% protein content, in addition to ad libitum wheat hay roughage. The ewes, originating from farms in Nablus and Tubas, range in age from 2 to 6 years, belong to a parity of 1 to 5, and weigh between 60 and 70 kg. Their diet includes a daily ration consisting of 1.5 kg of a concentrated mix with 16% protein content, in addition to 0.5 kg of wheat hay roughage. The protocols for all groups were carried out simultaneously.

The groups were not evenly distributed between the two farms; instead, each farm hosted a mix of ewes from all three groups to ensure a balanced representation of conditions across locations. The unequal division of ewes into groups (rather than three groups of 41) was intentional, based on the availability of resources and the specific objectives of the study, which required varying group sizes to test different insemination methods effectively.

### 2.3. Ethical Approval Statement

The protocols and animal manipulation in this project were approved by the Official Animal Care and Use Committee of the National Institute of Agronomy of Tunisia (Protocol No. 05/15).

### 2.4. Estrus Synchronization and Insemination

The ovarian response to synchronization schemes is influenced by various factors, including lactation. In this study, a synchronization scheme utilizing P4-lunga was employed, which eliminates the need for PGF administration. To counteract the inhibitory effects of lactation, a high dose of 600 IU eCG was administered. This approach ensures that the ewes can effectively respond to the synchronization protocol, optimizing reproductive outcomes.

Estrus synchronization in the ewes was facilitated by grouping them into three categories and administering intravaginal implant sponges (Chronogest/CR, Intervet, New Zealand) contains a concentration of 20 mg of fluorogestone acetate (FGA) for 12 days. Following the removal of the sponges, each ewe was given an intramuscular injection of eCG hormone at 600 IU. In the context of Assaf sheep in Palestine, the use of 600 IU of pregnant mare serum gonadotropin (PMSG) is common due to its effectiveness in enhancing reproductive performance during estrous induction protocols [25]. Additionally, lower doses may not provide the desired reproductive outcomes, particularly during the non-breeding season when hormonal activity is naturally reduced [26].

The administration of the eCG hormone to ewes was conducted via intramuscular injection. Initially, all equipment, including syringes and needles, was sterilized and prepared for use. The eCG hormone (Folligon, Intervet, The Netherlands), produced by CEVA, was then prepared following the manufacturer’s instructions, ensuring that a precise dosage of 600 IU was accurately drawn into the syringe for administration. After 36 h post-removal of sponges, ewes in Group 3 underwent initial natural mating. The subsequent mating took place nine hours later, employing the same ram. Ewes in Groups 1 and 2 were inseminated 48 h post-sponge removal using pooled fresh and frozen-thawed semen, respectively, adhering to the intracervical AI techniques outlined by Leethongdee [26]. The semen used was selected based on stringent criteria, requiring mobile spermatozoa score of over 60%, a sperm mass activity of at least 3, and a concentration of 30 × 10^6^ spermatozoa/mL.

### 2.5. Semen Preparation, Evaluation, and Insemination

In the experiment evaluating fresh and frozen ram semen for artificial insemination, the study applied global standards to assess quality [27]. For fresh semen, the study ensured a volume of 0.5 to 2 mL per ejaculation and achieved a sperm concentration of 2 to 6 billion per ml. High mass activity was observed, with total motility reaching at least 70% and progressive motility exceeding 30%. Circular motility was kept low, fast motility was high, and both slow and local motility were minimal, with a low percentage of immotile sperm. For frozen semen, the study noted the expected post-thaw reductions, with total motility at 40–50% and progressive motility at 20–30%, while addressing the challenge of immotile sperm exceeding 50%.

Semen collection and evaluation began on the same day as insemination and the initial assessment of the semen sample. Semen from 4 elite rams was collected using an artificial vagina (Ref.: 11320/0000, produced by Minitube), and, immediately after collection, the volume of the ejaculate was measured using a graduated cylinder or pipette to ensure accuracy, as CASA does not assess volume. For motility assessment, the CASA system, specifically the AndroVision^®^ from Minitube, Tiefenbach, Germany, was employed to evaluate both total and progressive motility, taking approximately 7 to 8 min. The semen sample was gently mixed to ensure uniformity and then loaded onto a pre-warmed microscope slide or chamber and examined under a microscope with a ×10 negative phase contrast objective on a 38 °C warmed stage (Eclipse E400, Nikon, Tokyo, Japan). The CASA system measures total motility, which is the percentage of moving sperm, and progressive motility, which is the percentage of sperm moving forward in a straight line. The sample was maintained at the correct temperature, typically 37 °C, during analysis to preserve sperm activity.

For concentration assessment, a fixed-height Minitube disposable counting chamber was used, as it is effective with CASA systems. The chamber is loaded with a well-mixed semen sample, ensuring no air bubbles are present, and then placed under the microscope connected to the CASA system. The system automatically counts the number of sperm in a defined area and calculates the concentration in terms of sperm per milliliter. All measurements, including volume, total motility, progressive motility, and concentration, are recorded accurately for analysis. These data are used to assess the quality of the semen sample, taking into account species-specific benchmarks and the intended use, such as artificial insemination. To prevent cold shock, the semen was maintained at 37 °C throughout the process. It was then diluted in a Tri’s egg yolk solution with glycerol at 30 °C and gradually cooled to 4 °C, over three hours [28]. A portion of this fresh semen was used for inseminating Group 1 ewes, while the remainder was frozen in 0.5 mL straws with liquid nitrogen (LN2). For Group 2, the frozen semen was thawed in a 37 °C water bath for 30 s before insemination. Each insemination dose contained 300 million sperm per straw. Group 1 ewes received fresh semen, and Group 2 ewes received thawed semen.

In the study, gestation diagnosis was established using a portable EasiScan 123 machine (BCF Technologies, Rochester, MN, USA) with ewes in the standing position. This method was performed 45 days after natural mating or artificial insemination to confirm pregnancy in the ewes. Reproductive parameters (prolific lambing and mortality rate) and lambs’ performances (live weight at birth and average daily gain) were recorded after lambing (142–155 days).

### 2.6. Deluent Preparation and Semen Freezing

Extender is made by dissolving tris-aminomethane (15.68 g), citric acid (8.76 g), lactose (14.11 g), and raffinose (25.4 g) in 1 L of sterile, double-distilled water. The solution is then sterilized by boiling it at 100 °C to eliminate any microorganisms. After the boiling process, the solution is allowed to cool down to room temperature (approximately 20–25 °C) before further use. After cooling, fresh egg yolk (20% of the total volume) 100,000 IU/100 mL penicillin, 0.1 g/100 mL streptomycin, and 13% glycerol are added.

After the semen is prepared, it is placed into a programmable temperature controller (TF-PA-II, Tianfeng Industrial, Shanghai, China) designed to precisely control the cooling process. This machine gradually lowers the temperature from 4 °C to −138 °C over 9 min. This gradual cooling is crucial to prevent thermal shock and ensure the viability of the sperm cells. Once the semen reaches the target temperature, it is transferred to a storage container filled with liquid nitrogen, where it is maintained at an extremely low temperature of approximately −196 °C. This cryogenic storage method preserves the semen for long-term use, maintaining its viability until it is needed for artificial insemination. When applying artificial insemination using frozen semen, the semen was subjected to a rapid thawing process to prevent ice crystal formation, which could harm the sperm cells. The frozen semen straws were immersed in a water bath at around 37 °C for about 30–60 s.

### 2.7. Insemination Technique

The cervical AI technique was applied by QuickLock insemination gun for small ruminants, to be used with Universal sheaths (Ref. 17350/0010), from minitube. It is considered the best technique for sheep due to its practicality and ease of use. This method involves depositing semen at the entrance of the cervix, which is less invasive compared to other techniques like laparoscopic AI. Cervical AI is advantageous because it can be performed quickly and with minimal equipment, making it accessible for many sheep producers. Additionally, it does not require the use of anesthesia or specialized surgical skills, reducing the stress on the animals and the cost of the procedure. Ewes were carefully restrained in a standing position within specially designed handling crates to ensure their safety and minimize stress during the procedure. This positioning allowed for optimal access to the reproductive tract while maintaining the comfort of the animals. Before the insemination process, the vulva of each ewe was thoroughly cleaned using a sterile solution (Chlorhexidine from Pfizer) and clean gauze pads. This step was crucial to prevent any potential contamination that could compromise the success of the insemination or lead to infections. Once the area was prepared, a speculum was gently inserted into the vaginal canal to provide a clear view of the cervix. The use of a speculum was essential for accurate placement of the semen, as it allowed the technician to navigate the anatomical structures with precision. Both groups of AI sheep from each farm were inseminated by the same technical person.

### 2.8. Statistical Analysis

Data were analyzed using SAS software version 9.4 (Statistical Analysis System, Release 9.4 2012 [29]. Data were tested for normality and variance under the assumption of homogeneity. All values were grouped, and the mean and standard error were calculated. A one-way analysis of variance using the general linear model was performed. Differences between LS means were assessed by SNK’s test. *p* < 0.05 was considered a significant value. Student’s test was used to compare two means. The following model was used:

Y_ijkm_ = μ + G_i_ + LS_j_ + Loc_k_ + Sex_l_ + e_ijklm_. (1)

where Y_ijklm_ refers to the observation; μ is the population mean; G_i_, the group effect (i = G1, G2, G3); LS_j_ effect of prolific lambing; Loc_k_ the effect of Location (k = Nablus, Tubas); Sexk (l = Male, Female); e_ijklm_ is the error.

Means of variables analyzed were compared using estimated statistical mean differences. Statistical differences were considered significant at *p* < 0.05.

## 3. Results and Discussion

### 3.1. Semen Characteristics

The semen characteristics of rams are essential for assessing their reproductive capabilities. Evaluating these characteristics is crucial for determining the reproductive health of rams, which supports the management of breeding programs and promotes favorable fertility outcomes [30,31]. Our research indicates that semen volumes in rams typically range from 0.8 to 2.5 milliliters per ejaculation, with sperm concentrations averaging between (2.9 × 10^9^) to (4.01 × 10^9^) sperm per milliliter, as shown in Table 1. Rams with sperm concentrations within the (0.8\times 10^9^) to (4.8 × 10^9^) spermatozoa per milliliter range are deemed normal, according to Jha et al. [31]. In terms of motility, ram sperm usually exhibit high progressive motility rates, often exceeding 68.3%, which is vital for successful fertilization. Our study’s sperm motility aligns with the findings of Svoradová et al. [32], who reported total motility and progressive motility (PM) rates of 81.12 ± 2.72% and 77.91 ± 3.15%, respectively. Ngcobo et al. [33] observed even higher total sperm motility in Zulu rams, ranging from 88.69 ± 1.40 to 92.01 ± 1.40%, with progressive motility between 50.86 ± 1.63 and 33.77 ± 1.63%. Notably, our findings reveal a higher percentage of progressively motile sperm (68.3%) compared to the 44% reported by Boshoff et al. [34]. The mass activity assessment, which gauges the coordinated movement of sperm cells, is also significant. Rams generally show robust mass activity scores (greater than 3), facilitating sperm transport within the female reproductive tract. Our results fall within the 2.4 ± 0.5 to 4.8 ± 0.4 range reported by Jha et al. [31]. We conclude that the semen quality of Palestinian Assaf sheep is adequately suited for use in artificial insemination (AI).

A review conducted by Ben Moula and El Amiri [35] highlighted the complex influence of seminal plasma (SP) on sperm function, affected by factors like breed and nutrition, without providing specific semen metrics. In contrast, the provided data detail semen volumes (0.8–2.5 mL) and high motility rates (>68.3%). While the review suggests SP impacts motility, the specific effects vary, aligning with the observed robust semen characteristics in rams. Understanding SP’s role is crucial for optimizing fertility outcomes in breeding programs.

### 3.2. Effects of Insemination Methods on Reproductive Performances: Conception Rate and Prolific Lambing

**Figure 1:**
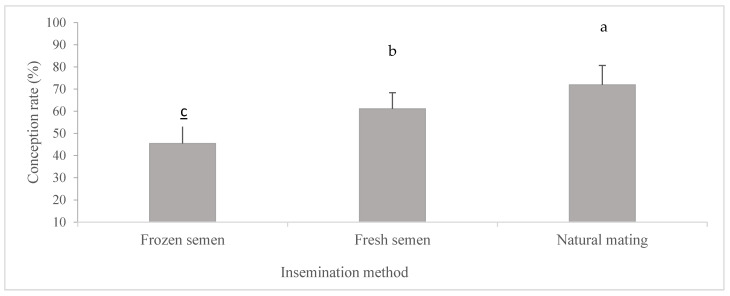
Conception rate under different insemination methods. Note: A different letter indicates significant differences (*p* < 0.05).

Several factors influence the reproductive performance of ewes, which varies according to the sexual season, breed, age, reproductive management (hormonal treatment or natural estrus), type of semen used in artificial insemination (AI) (fresh or frozen), AI technology (cervical or uterine), and the inseminator’s experience [36,37]. In this study, conception rates for pregnant individuals under different insemination methods were recorded in Figure 1. The results demonstrate a significant effect of insemination methods (sperm preservation) on conception rates (*p* < 0.05). There was a notable decrease in conception rates for ewes inseminated with fresh or frozen semen compared to those bred by natural mating. Specifically, the highest pregnancy rate was observed in Group 3 (G3) (71.9 ± 2.8; (*p* < 0.05)), representing natural mating, compared to the pregnancy rates in Groups 1 and 2 (G1 and G2) (61.1% ± 2.3 vs. 45.4% ± 1.9, respectively), which represent artificial insemination. Natural service in Kivircik ewes resulted in a significantly (*p* < 0.05) higher lambing rate and prolific lambing (86%; 2.0 ± 0.05 lambs/ewe) compared to artificial insemination (AI) using fresh (65%; 1.6 ± 0.1 lambs/ewe) or frozen (40%; 1.4 ± 0.14 lambs/ewe) semen. For AI animals, the lambing rate and litter size were not significantly different when service was performed at a fixed time (50%; 1.5 ± 0.12 lambs/ewe) or observed estrus (56%; 1.5 ± 0.12 lambs/ewe) [37]. Our results align with the commonly observed higher pregnancy rates in natural mating scenarios. Research has indicated that natural mating generally results in higher pregnancy rates compared to artificial insemination (AI) using fresh or frozen semen. Studies have shown that, while AI could be beneficial, particularly for subfertile animals, the success rates often fell short of those achieved through natural mating. Various investigations highlighted that fresh semen insemination yielded better outcomes than frozen, reinforcing the preference for natural mating in many breeding scenarios [1,27,38,39,40]. Hiwasa et al. [41] found that intrauterine artificial insemination (AI) with frozen-thawed semen using the AndroMed extender achieved a fertilization rate of 81.0% and a pregnancy rate of 72.2%, both significantly higher than cervical AI methods. In contrast, cervical AI with fresh-diluted semen resulted in a fertilization rate of 25.5% and a pregnancy rate of 5.5%, while frozen-thawed semen yielded a fertilization rate of only 3.5% and a pregnancy rate of 0.0%. Conversely, Hackett et al. [38] found no significant differences in reproductive performance between ewes subjected to natural mating and those that underwent artificial insemination (AI) using semen collected by either an artificial vagina (AV) or electroejaculation (EE). Both methods demonstrated comparable success rates, with 45% for natural mating and 30% for AI. Furthermore, no significant difference was observed between the two semen collection methods, with success rates of 30% and 29%, respectively. However, fertility was significantly higher (*p* < 0.05) in ewes treated with pregnant mares’ serum gonadotrophin (PMSG), achieving a success rate of 33% compared to 11% in untreated ewes. This increase in fertility was mainly attributed to higher fertility in ewes undergoing AI applications. Furthermore, PMSG administration significantly impacted prolificacy and fecundity (*p* < 0.01), with values of 1.8 and 58%, respectively, compared to 1.5 and 16% in untreated ewes. Fukui et al. [39] conducted research that highlighted the significance of sperm number per dose and ewe age on the fertility of Suffolk ewes after intrauterine insemination with frozen semen, concluding that these factors are crucial in determining fertility outcomes in this reproductive technique. Masoudi et al. [21] observed that natural mating consistently resulted in higher pregnancy and lambing rates compared to artificial insemination (AI). Additionally, this study highlighted the advantages of hormonal treatments in improving fertility, a factor that was not addressed in the Masoudi research. Both studies concur that natural mating typically yields better results than AI.

This study highlights key factors influencing reproductive performance in Palestinian Assaf sheep, such as breed, age, reproductive management, semen type, and the technology employed in artificial insemination. It shows that natural mating yields higher conception, lambing rates, and prolific lambing compared to AI with fresh or frozen semen. Hormonal treatments like eCG significantly enhance fertility, prolificacy, and fecundity in ewes. Optimizing AI methods and considering factors like sperm dose and ewe age are crucial for improving AI success. Addressing these elements can enhance the productivity and sustainability of sheep farming in the region.

**Figure 2:**
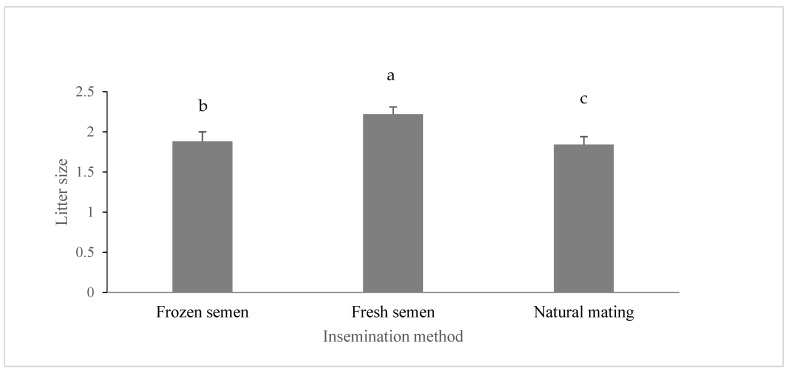
Effect of insemination methods on prolific lambing. Note: A different letter indicates significant differences (*p* < 0.05).

The effect of the insemination method on prolific lambing is presented in Figure 2. Prolific lambing was significantly affected (*p* < 0.05) by the insemination method, with the highest value recorded in ewes inseminated with fresh sperm. Donovan et al. [42] showed that prolific lambing was affected by semen type (fresh or frozen) due to the increased adverse effect of frozen semen on prolific lambing in synchronized ewes (*p* < 0.05). The same authors demonstrated that the pregnancy rate was significantly influenced by ewe breed (*p* < 0.01) and inseminator (*p* < 0.05). These results suggest that the ewe breed may be a critical determinant for the exploitation of cervical insemination of frozen-thawed semen in sheep breeding programs. Additionally, nutritional flushing resulted in a higher number of lambs born to control ewes. Najafi et al. [43] revealed that twinning rates ranged from 10 to 34.6%, and prolific lambing varied from 1.1 to 1.35, with significant differences observed between the treatment groups and the control group (*p* < 0.05). Olesen and Ingrid [44] found that elite Norwegian ewes that were artificially inseminated had a smaller prolific lambing at birth, averaging about 0.2 to 0.4 fewer lambs compared to those that were naturally mated. It is important to note that this difference in prolific lambing is not solely due to the artificial insemination (AI) method itself, but rather because AI utilizes genetically superior rams. Despite this, AI remains an effective method for spreading enhanced genetic traits in Norwegian sheep.

The results of this study were obtained during the spring of 2022, a period out of season for sheep breeding in Palestine, which typically occurs from late July to early December. This timing is significant, as it reflects the challenges associated with breeding outside the natural estrus cycle of ewes, which can lead to lower conception rates and reproductive performance. In this study, hormonal treatments were employed to induce estrus in the ewes during this off-season period, specifically using an intramuscular injection of 600 IU of PMSG (Pregnant Mare Serum Gonadotropin) following the removal of intravaginal sponges. Research indicated that 600 IU of PMSG is sufficient to induce a strong estrous response while minimizing potential side effects associated with higher dosages. For instance, studies have shown that, while increasing the dose of PMSG can enhance ovulation rates and prolific lambing, the benefits tend to plateau at around 600 IU, making it a practical choice for sheep. Additionally, lower doses may not provide the desired reproductive outcomes, particularly during the non-breeding season when hormonal activity is naturally reduced [44,45]. Despite these challenges, the findings of this study demonstrate that artificial insemination, particularly with fresh semen, can still yield significant improvements in lamb growth performance, highlighting the potential for effective reproductive management strategies even during non-breeding seasons.

### 3.3. Effect of Insemination Methods on Lambs’ Growth Performance

Table 2 showcases the performance of lambs subjected to various insemination methods. The study’s findings highlight a significant impact of the insemination method on lamb growth performance (*p* < 0.05). Specifically, the birth weights of lambs in groups G1 and G2 showed a significant increase (*p* < 0.05) with weights of 4.011 kg and 4.208 kg, respectively, compared to 3.468 kg in group G3. This contrasts with the conclusions of Geenty et al. [46], who found the reproductive rate in an artificial insemination (AI) program to be on par with natural mating. Notably, optimal survival rates for lambs are associated with birth weights within the 4–8 kg range.

Additionally, Fogart et al. [47] observed that using thawed-frozen semen for artificial insemination (AI) in ewes significantly influenced birth weight, which varied based on ram breed. The birth weights ranged from 4.0 to 4.4 kg, with an average prolific lambing of 1.8 lambs. This study also revealed that the method of insemination significantly affected (*p* < 0.05) the weaning weight of lambs at 60 days, with recorded weights of 21.11 kg for Group G1, 20.38 kg for Group G2, and 18.54 kg for Group G3. Furthermore, Fogarty et al. reported that the ram breed used in AI with thawed-frozen semen significantly influenced weaning weight, which ranged from 19.6 to 22.5 kg. The study further demonstrated a significant effect (*p* < 0.05) of the insemination method on the final weight of lambs at 180 days and their average daily gain. The recorded weights for lambs in Groups G1, G2, and G3 were 65.7 kg, 67 kg, and 60.44 kg, respectively, while the average daily gains were 0.284 kg, 0.293 kg, and 0.251 kg, respectively. All these findings are attributed to the research conducted by Fogart et al. [47].

Figure 2 presents the results of the effect of the insemination method on prolific lambing, as there is no significant difference (*p* > 0.05) in the newborns’ weight, weaning weight, and marketing weight between the three groups. The birth weights from the first prolific lambing to the fourth prolific lambing were as follows: 4.4 ± 0.21, 4.1 ± 0.61, 3.8 ± 0.33, and 3.3 ± 0.65 kg, respectively. The weaning weight was 21.5 ± 0.29, 20.3 ± 0.17, 19.9 ± 0.37, and 18.3 ± 0.7 kg, respectively. The marketing weight at 180 days was as follows: 64.79 ± 0.62, 66.48 ± 0.33, 64.38 ± 0.7, and 63.9 ± 1.4 kg, respectively.

### 3.4. Effect of Insemination Method and Lamb Sex on Growth Performance

Table 3 details the effects of the insemination method and lamb sex on the weight of lambs at different developmental stages. This study demonstrates that both the insemination method and the sex of the lamb significantly affect (*p* < 0.05) the weaning weight, the average daily gain from 60 to 180 days, and the final weight at 180 days. According to the table, male and female lambs conceived with fresh semen had birth weights of 4.31 kg and 4.16 kg, respectively, which were greater than those of male and female lambs from natural mating, with weights of 3.59 kg for males and 3.29 kg for females. Male lambs from natural mating were heavier than females by 0.3 kg. For lambs conceived with frozen semen, the weight difference was 0.35 kg for males and 0.8 kg for females compared to those from natural mating. At 60 days, lambs from fresh semen were heavier in both sexes. Lambs from frozen semen also weighed more in both sexes compared to those from fresh semen and natural mating, with differences of 0.15 kg and 2.53 kg for males, and 1.13 kg and 2.5 kg for females. At 180 days, lambs from fresh semen were heavier in both sexes, with differences of 1.45 kg and 5.77 kg for males, and 1.38 kg and 7.46 kg for females. The average daily gain at 60 days for male and female lambs from natural mating was similar across all mating methods (*p* = 0.194). Ilić et al. [48] and Yilmaz and Altin [49] observed that mating methods and lamb sex significantly influence birth weight, a key factor in lamb production, corroborating our findings. Mating methods also impacted the body weight and average daily gains of lambs from birth to 180 days, in line with the findings of Petrovic et al. [50].

## 4. Conclusions

This study explored the reproductive performance of lamb growth in Palestinian Assaf sheep using natural mating and artificial insemination (AI) with fresh and cryopreserved semen. Natural mating yields the highest fertility rates, while AI, especially with cryopreserved semen, enhances lamb growth indicators like birth and weaning weights. Prolific lambing affected early growth, and management practices and lamb sex influence growth rates. To improve genetic quality, AI with superior rams is recommended, despite lower fertility rates. Breeding programs should balance natural mating for fertility and AI for genetic gains. By understanding the impact of different breeding methods on reproductive performance and growth traits, this study provided valuable insights for sheep farmers and researchers to optimize breeding strategies and improve livestock productivity in Palestine. Moreover, the breeding season for Palestinian Assaf sheep typically extends from late July to early December, during which ewes naturally exhibit estrus. To enhance breeding opportunities outside this natural period, hormonal treatments, such as eCG (Folligon^®^), were effectively utilized to induce estrus. Further research is needed to assess long-term impacts on productivity and economics.

## Figures and Tables

**Table 1:** Evaluation of fresh and frozen semen used in artificial insemination.

	VL ml	Conc. (10^9^/mL)	MS (0–5)	TM (%)	PM (%)	CM (%)	FM (%)	SM (%)	LM (%)	Im (%)
Ram (Fresh Semen)										
36	0.8	3.04	3	83.3	64.2	0	28.8	35.4	19.2	16.7
46	1	3.15	3	86.4	64.6	0.2	55.7	8.7	21.8	13.6
48	2.5	2.81	3	87.5	68.3	0.6	56.1	11.6	19.2	12.5
52	2.3	3.2	3	80.7	60.3	0.3	43.9	16.1	20.4	19.3
Frozen Straw										
36			3	42.5	26.3	0	8.6	17.7	16.2	57.5
46			3	43.7	24.1	0	3.9	20.2	19.6	56.3
48			3	42.9	25.5	0	11.1	14.4	17.4	57.1
52			3	44.5	23	0	1.9	21.6	20.7	55.5

VL: Volume; Conc.: Sperm Concentration; MS: Mas Activity; TM: Total Motility; PM: Progressive Motility; CM: Circular Motility; FM: Fast motility; SM: Slow motility; LM: Local motility; Im: Immotile.

**Table 2:** Variation in lamb growth performance according to insemination methods.

Factor	Insemination Methods		
	^1^ G1 (*n* = 25)	G2 (*n* = 57)	G3 (*n* = 38)
Birth weight (Kg)	4.01 ^a^ ± 0.22	4.21 ^a^ ± 0.15	3.47 ^b^ ± 0.19
Weaning weight at 60 days (Kg)	21.12 ^a^ ± 0.32	20.38 ^a^ ± 0.22	18.55 ^b^ ±0.29
Final weight at 180 days (Kg)	65.7 ^a^ ± 0.65	67.1 ^a^ ± 0.42	60.4 ^b^ ± 0.63
Average daily gain at 60 days (Gram)	0.282 ^a^ ± 0.65	0.269 ^a^ ± 0.65	0.239 ^b^ ± 0.65
Average daily gain at 180 days (Gram)	0.284 ^a^ ±0.03	0.293 ^a^ ± 0.64	0.251 ^b^ ± 0.95

^1^ G1: Group 1: frozen semen; G2: Group 2, fresh semen; G3: Group 3, natural mating. a and b indicate the significant differences between the groups at the threshold of 5%.

**Table 3:** Effect of lamb sex on growth rate performance in the three groups.

Factor	Sex–Lamb							
	Female				Male			
	G1	G2	G3	Std. Error	G1	G2	G3	Std. Error
Number	12	39	16		13	18	22	
BW (Kg)	4.09 ^a^	4.16 ^a^	3.29 ^b^	0.181	3.94 ^b^	4.31 ^a^	3.59 ^b^	0.172
WW (Kg)	20.802 ^a^	19.668 ^b^	18.303 ^c^	0.242	21.334 ^a^	21.179 ^a^	18.797 ^b^	0.236
FW (Kg)	63.308 ^a^	64.780 ^a^	59.011 ^b^	0.488	67.994 ^a^	69.379 ^a^	61.915 ^ab^	0.466
ADG60	265.761 ^a^	265.840 ^a^	265.982 ^a^	4.034	265.785 ^a^	265.857 ^a^	265.751 ^a^	3.928
ADG180 (Gr)	275.434 ^a^	275.362 ^a^	275.338 ^a^	3.045	275.277 ^a^	275.223 ^a^	275.376 ^a^	2.910

BW: Birth Weight; WW: Weaning weight at 60 days; FW: Final weight at 180 days; ADG60: Average daily gain at 60 days; ADG180: Average daily gain at 180 days; G1 = Group 1, frozen semen; G2 = Group 2, fresh semen; G3 = Group 3, natural mating. Note: A different letter indicates significant differences (*p* < 0.05).

## Data Availability

The data sets generated during the current study are available from the corresponding author upon reasonable request.
